# Seed treatment: an alternative and sustainable approach to cotton seed delinting

**DOI:** 10.3389/fbioe.2024.1376353

**Published:** 2024-08-29

**Authors:** Laviza Tuz Zahra, Fariha Qadir, Mohammad Nasir Khan, Hira Kamal, Nosheen Zahra, Arfan Ali, Muhammad Mubashar Zafar, Abdul Razzaq, Xuefei Jiang

**Affiliations:** ^1^ Institute of Molecular Biology and Biotechnology, The University of Lahore, Lahore, Pakistan; ^2^ Renewable Energy and Environmental Technology Center, University of Tabuk, Tabuk, Saudi Arabia; ^3^ Department of Plant Pathology, Washington State University, Pullman, WA, United States; ^4^ FB Genetics Four Brothers Group Pvt. Limited, Lahore, Pakistan; ^5^ Sanya Institute of Breeding and Multiplication, Hainan University, Sanya, China; ^6^ School of Tropical Agriculture and Forestry, Hainan University, Danzhou, China

**Keywords:** cotton, delinting, sustainable development goals, biological delinting, chemical delinting

## Abstract

This review article delves into the vital aspects of cotton, emphasizing its global significance as a crucial agricultural commodity. The paper comprehensively explores the composition of cotton and surveys the diverse methods employed for the removal of cotton lint from seeds. Conventional delinting methods, including mechanical and chemical approaches, are scrutinized in terms of their advantages and drawbacks. However, the primary focus of this review is on highlighting the emerging significance of biological delinting methods. By harnessing the power of microbial enzymes and organisms, biological approaches offer a promising alternative for efficient lint removal. The authors discuss the environmental advantages associated with biological delinting, positioning it as a sustainable solution that mitigates the ecological impact of traditional methods. Furthermore, the article contextualizes these delinting methods within the framework of Sustainable Development Goals (SDGs) and underscores the importance of adopting eco-friendly practices in the cotton industry to align with SDG goals. By accentuating the potential of biological delinting in contributing to sustainable agriculture and responsible production, the review advocates for a paradigm shift towards more environmentally conscious approaches in the cotton sector. Overall, the article aims to provide a comprehensive perspective on cotton delinting methods, emphasizing the pivotal role of biological alternatives in fostering a sustainable and goal-oriented future for the cotton industry.

## Introduction

Cotton also referred to as white gold in various regions of the world, is a crop that contributes significantly to the world’s economy, producing global income for over 250 million people (Munir et al., 2020; Arshad et al., 2022; Zhao et al., 2023). Amongst other agronomic crops, cotton enjoys a particular distinction, owing to its perennial growth and fruiting habit (Maeda et al., 2021). The coat of cottonseed can develop into cellulose-rich fibers while on the other hand, the embryo yields proteins and oils, attributing high economic value to both maternal and filial tissues of cottonseed (Maeda et al., 2021; Khan et al., 2020). *Gossypium* genus holds claim over more than 50 species found within a range from arid to semiarid regions of the world (Razzaq et al., 2021). The genus came into existence nearly 10–15 million years ago and currently boasts eight major genome groups (A through G and K) for diploids. In the last 1–2 million years due to the hybridization between A and D genome diploid, where the former was subjected to transoceanic dispersal, allopolyploid cotton appeared (Chen et al., 2020). Four cotton species are known to be grown commercially in which *Gossypium hirsutum*, a tetraploid, is also referred to as upland cotton since it is the most popular choice for cultivation worldwide. *Gossypium barbadense* is next in choice, offering high staple length. The other two species, both diploids in nature, are *Gossypium arboreum* and *Gossypium herbaceum* (Iqbal et al., 2023) (Hu et al., 2021 *)*.

In terms of botany, cotton fiber is understood to be a unicellular trichome that arises from the outer layer of the seed coat (Naoumkina and Kim, 2023; Sarwar and Iqbal, 2020; Xiao et al., 2019; Kim, 2015). Not unlike other plant cells, the development of cotton fiber is sculpted by the variations in the delicately intertwined micro and macro environments. Natural fiber can fairly be described as a composition of cellulose microfibrils with several other chemical composites found in minor presence. The composition is prone to change by the environment and the growth rate of the plant, in addition to the degree of maturity (Liyanage and Abidi, 2019). However, an average mature fiber carries (88.0%–96.5%) cellulose, with noncellulosic constituents such as proteins (1.0%–1.9%), waxes (0.4%–1.2%), pectins (0.4%–1.2%), inorganics (0.7%–1.6%), and other substances (0.5%–8.0%). Pure cellulose is predominantly found in the secondary cell wall (SCW) region, while noncellulosic constituents coexist either on the outer layers (cuticle and primary cell wall, PCW) or within the fiber lumens (Liu, 2018; Zubair et al., 2021).

The development of cotton is a complex process that can be divided into four stages, showing considerable overlap. The first stage, initiation, occurs between 3 days before anthesis and 2 days after anthesis, with some researchers suggesting it may extend beyond 5 days after anthesis (Xiao et al., 2019; Hu et al., 2018; Kljun et al., 2014). During this stage, fibers of approximately 2.2–3.6 cm length undergo differentiation within the ovary of the flower, known as the cotton boll (Kim, 2015; Wang et al., 2021). Following initiation, fibers elongate rapidly and longitudinally until 20 DPA, reaching a final length of 22–35 mm and depositing a thin primary cell wall (PCW) layer (Qin et al., 2022). At approximately 14–16 DPA, fibers enter the secondary cell wall (SCW) stage, ceasing the synthesis of other cell wall polymers and dramatically increasing cellulose synthesis. Fiber elongation continues until approximately 21–26 DPA, with the PCW biosynthesis process closely related to proteins, pectins, fatty acids, calcium ions, and sugars (Liu, 2018; Sun et al., 2023). During the maturation phase, cotton bolls reach their maximum weight and size. In this stage, secondary wall thickening of fibers occurs, with active synthesis of cellulose in the fiber and oil in the embryo (Li et al., 2018). The cellulose increases quickly during this transition, forming ordered arrangements of pure cellulose microfibrils helically oriented along the growing fiber (Lu et al., 2022; Liu, 2018). Sucrose translocated from leaves serves as the energy source for biosynthesis, moving inward for the inner seed coat and embryo and outward for fibers. Inward translocation is used for synthesizing lipids and proteins in the embryo (Tariq et al., 2020). According to mature fiber length, cotton fibers are categorized into lint and fuzz. Lint fibers initiate from 0 DPA to 3 DPA, while fuzz fibers initiate from 5 DPA to 10 DPA (Hu et al., 2018).

The cotton seed consists of the embryo, endosperm, perisperm, inner pigment layer, palisade (Malpighian) layer, colorless layer, outer pigment layer, and epidermis including lint hairs (Reeves and Valle, 1932). A mature cottonseed embryo itself contains the radicle, hypocotyl, a primordial epicotyl, and two cotyledons (Maeda et al., 2021). The seed of the cotton plant can be considered one of the most coveted parts since it offers advantages to numerous industries including textile, medicinal products, animal feed, paper, and edible oil (Ali et al., 2020). This review discusses the various methods employed for cottonseed delinting (chemical, mechanical and possible biological techniques), detailing the processes, advantages, and potential drawbacks of each method. Remarkably, various chemical components can be harvested from cottonseed depending on the maturity of the seed. Proteins obtained from cottonseed have also exhibited remarkable adhesive properties (Cheng et al., 2016; Kumar et al., 2021). As a seed grows in age, the concentration of oil and protein it carries also appears to increase, while the opposite is true for the concentration of starch which declines as the seed grows older. During the developmental stages of the cottonseed, maltose reigns supreme in concentration (Tariq et al., 2020). Despite all the advancements cotton production has seen, lack of seed quality is an issue that still arises from occasion to occasion and endangers fiber quality and crop yield (Atique-ur-Rehman and Afzal, 2020).

## Cotton lint degradation

In a process referred to as ginning, seeds are separated from cotton and debris. However, regardless of the efficiency of this process, a layer of lint (or fuzz) remains on the surface of the cottonseed. This fuzz, although harmless in appearance, can prove to be detrimental to the characteristics and activities of the cottonseed in the processes after ginning (Atique-ur-Rehman and Afzal, 2020). This cottonseed lint can obstruct the sowing and planting of the seed, which will ultimately lead to a setback in the crop quality and yield. Study showed that delinted cottonseeds exhibited a higher germination percentage of 90%, whereas seeds with lint had a lower percentage of 82% (Brown, 1933). This was further confirmed by other research groups as well (Maeda et al., 2021; Nowrouzieh et al., 2024). Another more harrowing effect of the presence of lint on cottonseed is its ability to retain water, which would breed fungal infections (Afzal et al., 2020) (Heydari, 2005). Research highlighted that delinted seeds showed approximately 25% reduction in disease incidence compared to non-delinted seeds treated with fungicides and insecticides (Heydari, 2005). With that being established, it is imperative to remove the lint found on the surface of the cottonseed, to maintain the quality of the seed physiology and ensure high performance (Holt et al., 2017; Lima et al., 2023).

The term that encapsulates the processes involved in the removal of the aforementioned fuzz is “delinting”. It carries types from the popular mechanical and chemical to the less renowned thermal and biological, delinting.

## Cotton lint degradation approaches

### Chemical delinting

Amongst all the types of delinting methods, chemical delinting is practiced most widely. The chemical procedure consists of cottonseed being subjected to sulphuric acid treatment (Atique-ur-Rehman and Afzal, 2020). The treatment has been performed with concentrated and dilute sulphuric acid both with little to no significant difference in the results. Sulphuric acid (98%) acts as an oxidizing agent that facilitates the degradation of tissues and organic compounds, especially cellulose. Therefore, after the removal of the lint from the cottonseed is achieved, the process is followed by washing and a particular step referred to as neutralization (Queiroga and Mata, 2018; Dowd et al., 2019; Maeda et al., 2021)). This step is intended to halt any further processions of the acid, preserve the physiological health of the seed, and prevent any storage and environmental issues. This method enjoys certain distinction amongst other methods since not only are the involved reagents cheap, but they have also proven themselves to be highly effective in the degradation of cotton lint. The acid treatment in tandem with the subsequent step of washing, has yielded favorable results by increasing the water content of the seed (de França et al., 2018). Despite all of its acclaim, chemical delinting has significant drawbacks which categorically stem from the use of acid (Zhou et al., 2015). As mentioned before, sulfuric acid is an oxidizing reagent and it stands to reason that if any hint of residue remains, it can damage not only the seed but also the environment in which the seed was stored or sown. It was found that the pH of liquid effluent was 0.60 (Tostes et al., 2023). Given how crucial this is, it is astounding to note that although numerous resources have been devoted to refining the acid treatment, the neutralization step has not garnered enough attention and research (Dowd et al., 2019; Lima et al., 2023). Currently, different bases (sodium hydroxide, sodium carbonate, and calcium hydroxide) are used to perform neutralization but these activities generate effluents (Table 1) that must be suitably disposed of (Afzal et al., 2020; Tostes et al., 2023). Unfortunately, a well-established standard for neutralization and its effluent disposal does not yet exist and breeds questions about an otherwise commendable method (Tostes et al., 2023).

**TABLE 1:** Mean pH of residual liquid after delinting and neutralization process (Tostes et al., 2023).

Neutralizing agent	pH
Sodium hydroxide	1.30
Sodium carbonate	1.17
Calcium hydroxide	1.70

### Physical delinting

#### Mechanical delinting

**FIGURE 1:**
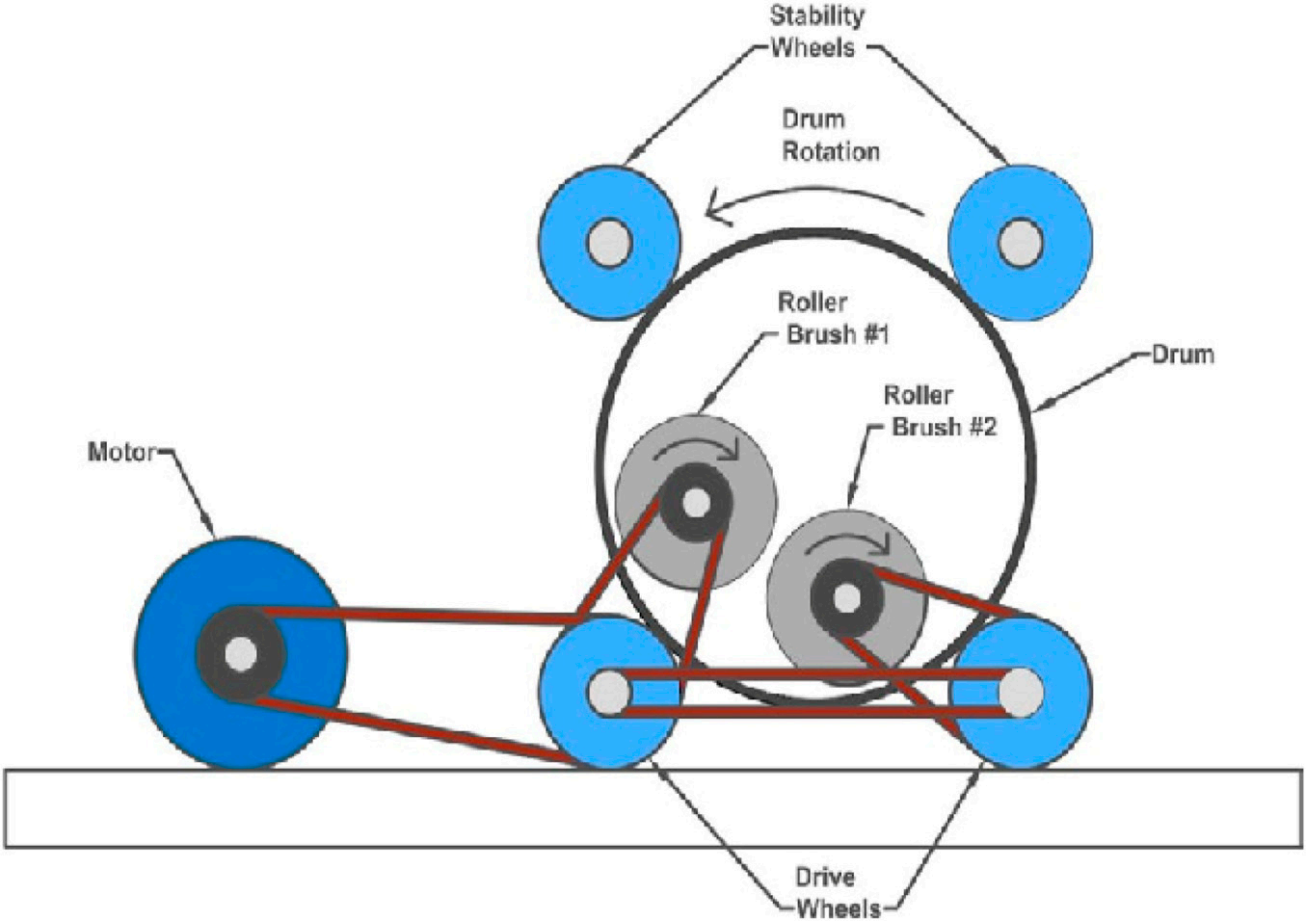
Illustration of a benchtop cottonseed delinter (Holt et al., 2017).

Mechanical delinting is one of the earliest methods of delinting cotton seeds that is achieved through the use of varyingly elaborate machinery. The principal activity behind mechanical delinting of cottonseed involves the seeds’ fiber ends being pulled by the motion of rotating cylinders or rollers, while the cottonseed is firmly held in place. The precise method of each model of machine is subject to change where some models contain multiple rotating cylinders to enhance efficiency while other machines involve subsequent cycles of the same process to ensure that the cottonseed is fully delinting (Ugwu et al., 2015; Zhou et al., 2015). Figure 1 presents a schematic of a benchtop cotton seed delinter (Holt et al., 2017).

Owing to the lack of chemicals used in mechanical delinting, this method rightfully boasts a certain advantage over other delinting methods, especially chemical delinting which attributes its function to acid as summarized in Table 2 (Holt et al., 2017; Zhou et al., 2015). Mechanical delinting does not require any post-delinting steps such as neutralization of acid as mentioned above in chemical delinting (Tostes et al., 2023). Since the fuzz from the cottonseed did not undergo any chemical treatment, it provides a distinct opportunity to use the lint, thus proving itself to be an environmentally supportive method (Afzal et al., 2020; Zhou et al., 2015). Mechanical delinting as a method, however, is not void of shortcomings. While performing mechanical removal of lint from cottonseed, the seed coat can suffer damage if the abrasive forces are not appropriately managed, and measures to maintain temperature are necessary to protect the seed. Furthermore, despite the simplicity of the principle of the machinery, the operation itself is precarious and can inflict heavy damage if not optimized carefully. It is also worth noting that the cost and energy required to manage the machinery are very high and harbor a set of environmental concerns (Zhou et al., 2015).

**TABLE 2:** Comparison of mechanical and chemical delinting

Aspect	Mechanical delinting	Chemical delinting (sulfuric acid)
Environmental Impact	No chemical waste but energy consuming	Produces acidic effluents that require neutralization
Seed Quality	High abrasion and temperature can damage seed	Effective lint removal but potential for seed coat damage if not carefully managed
User Safety	No chemical exposure but heavy machinery can be hazardous	Requires handling of corrosive acid, posing health risks to operators
Process Efficiency	High throughput, less time-consuming	Effective but requires additional steps for neutralization and effluent management
Operational Complexity	Varying models of machinery. Not always easy to operate	Requires careful handling and disposal of hazardous chemicals

#### Thermal delinting

Another method joining the ranks of cottonseed delinting is referred to as thermal delinting. Although it is not as highly celebrated as the mechanical and chemical delinting methods, thermal delinting has been explored as a viable option for cotton lint removal. The most pronounced work currently found on thermal delinting is by (de França et al., 2018) who subjected the lint on the surface of cottonseed to flames of varying intensities, by employing a thermal deliniter prototype. Several aspects of this method are worth rumination. The period for which the cottonseed is exposed to heat is incredibly crucial since a longer exposure can be detrimental to the physiochemical characteristics of the seed, especially to the water content (de França et al., 2018). Thermal delinting is not preferred over the other existing delinting methods since it entails high energy consumption and breeds environmental concern over gas emissions.

#### Biological delinting

Given how all of the current methods come at the expense of either the environment or the health of the cottonseed, the authors propose an alternative method that would ensure that no harm comes to the environment and the cottonseed is successfully delinted while potentially maintaining its quality. The alternative method should explore biological moieties for delinting such as cellulase enzyme.

In the past, the digestion of cotton by cellulolytic enzymes of *Fibrobacter succinogenes* was carried out, notably by (Palmquist, 1995). In two sets of experiments, the rumen microbial digestion of whole oilseeds was assessed in sacco (Experiment I), and the digestion of cotton lint was evaluated both in sacco and *in vitro*. In the second experiment, cotton linters underwent incubation in sacco and *in vitro* over periods ranging from 12 to 120 h. Notably, digestibility at the 12-h mark was close to zero, despite microscopic observations confirming microbial colonization of the fibers. Digestibility displayed a linear increase from 12 to 72 h of incubation, indicating a limitation in cellulolytic activity. Pre-soaking the fibers in distilled water for 24 h before incubation significantly enhanced digestibility (*P* < 0.0001). Ether extraction before incubation not only improved digestibility but also appeared to have an additive effect when combined with wetting. The soaking of fibers in 4% NaOH for 24 h had a variable positive impact on digestibility. The delay in the digestion of cotton fibers after colonization was attributed to the highly crystalline structure of the fibers, which hydrates slowly. Ether extraction was effective in removing a hydrophobic layer, facilitating more rapid water penetration. Alkali treatment, however, did not alter the crystallinity of cotton fibers. The lag in the digestion of cotton fibers was seen as a factor that could increase the pool size of undigested fiber in ruminants consuming whole-lined cottonseed. The delayed digestion was hypothesized to be a result of the time required to hydrate the highly crystalline cellulose before cellulolysis could proceed (Palmquist, 1995).

Another group of scientists delved into the characterization of microbial species involved in the degradation of cellulose and hemicellulose in animal intestines. A significant substrate for fermentation in the large intestine is fiber and in pigs, cellulolytic organisms such as *Bacteroides succinogenes* and *Ruminococcus flavefaciens* are present in numbers comparable to those in the rumen. Given that other conditions are satisfied, there exists substantial potential for fiber degradation in the large intestine of pigs (Varel, 1987).

In the last decade, in the textile industry, commercial cellulose gained popularity to conduct a process called biopolishing. Commercial cellulase was successfully immobilized onto Concanavalin A (Con A)-layered calcium alginate beads, resulting in the cellulase retaining approximately 82% of its initial activity post-immobilization (Sankarraj and Nallathambi, 2015). Then in further investigations, free and immobilized cellulase was employed for the biopolishing of cotton fabric. The immobilization process enhances the biopolishing effectiveness while mitigating the loss of physical properties. Through the cellulase enzyme’s hydrolysis during the biopolishing process, changes occur in the cotton fibers’ tensile strength, fabric weight, and whiteness index. The reduction in tensile strength is minimized when employing immobilized cellulase compared to the free form (Sankarraj and Nallathambi, 2018).

**FIGURE 2:**
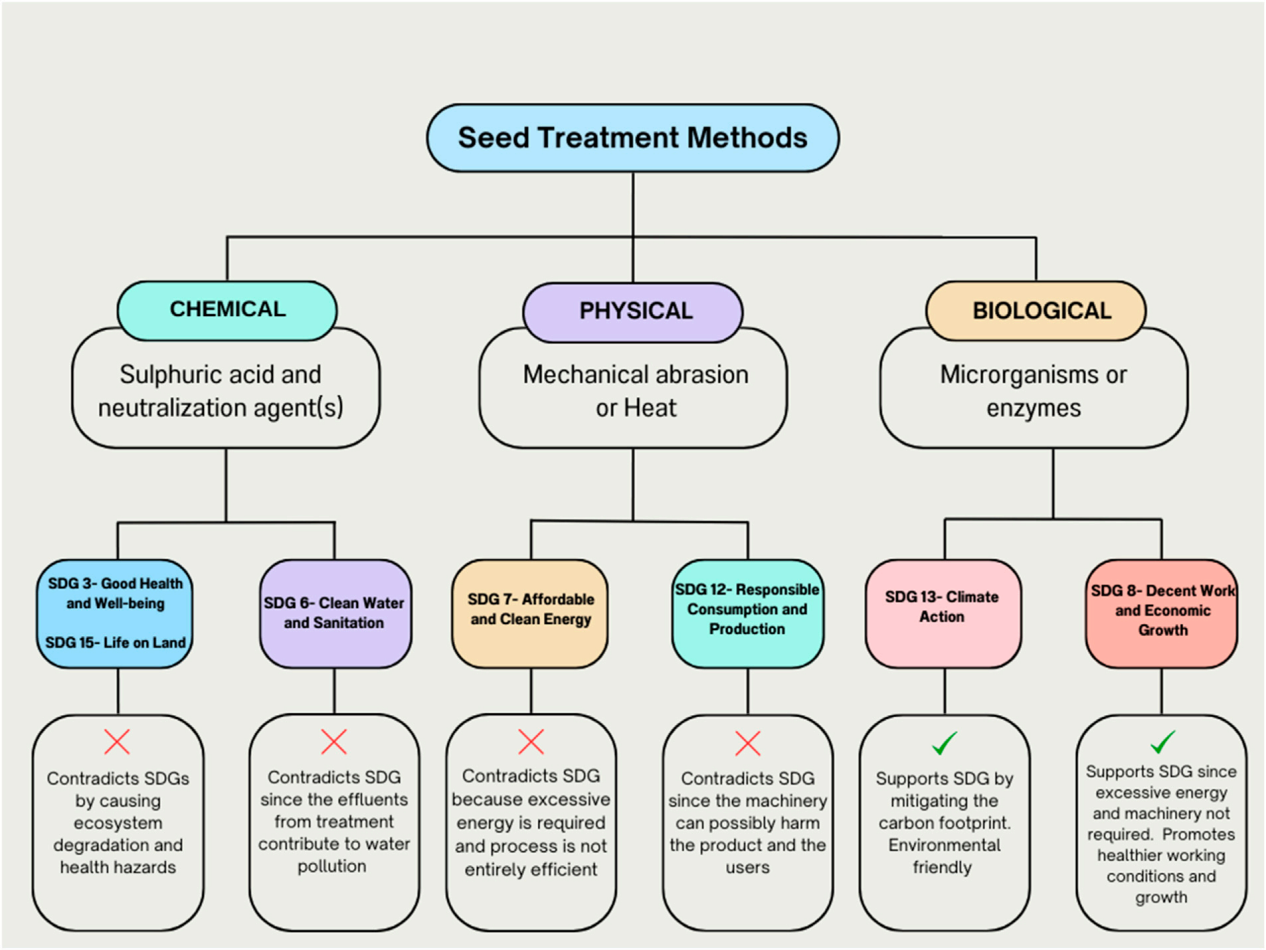
Seed treatment methods and their direct relationship with the SDGs.

Biological delinting of cottonseed, while promising, requires extensive exploration and research to fully understand its impact on seed quality and performance. However, the authors’ suggestion for employing biological methods of delinting over chemical and physical alternatives stems from the inherent environmental and sustainable advantages offered by the former. Biological delinting methods, utilizing microorganisms or enzymes, contribute to a reduced ecological footprint by minimizing the use of harsh chemicals and energy-intensive processes. Unlike chemical method that generates acidic waste (pH 1.17-1.70) as determined by (Tostes et al.,2023) that poses potential hazard, biological approaches are inherently more eco-friendly. Moreover, biological delinting processes are often milder on the cotton fibers, preserving their quality and enhancing the overall efficiency of downstream processing. Embracing biological delinting aligns with the global shift towards sustainable practices, emphasizing a greener and more responsible approach in the crucial domain of cotton processing. Different seed treatment approaches with reference to Sustainable Development Goals (SDGs) are listed in Figure 2.

## Cotton delinting and SDGs

Chemical and physical delinting methods often run counter to the Sustainable Development Goals (SDGs) due to their environmental and social implications. Chemical delinting frequently involves the use of harsh substances that may lead to water pollution, soil degradation, and health hazards for both workers and local communities. This contradicts SDG 3 (Good Heath and Weel-being), SDG 6 (Clean Water and Sanitation) and SDG 15 (Life on Land) by contributing to water pollution and ecosystem degradation. Physical delinting methods, such as abrasive processes, can be energy-intensive and may lead to excessive water usage. This goes against SDG 7 (Affordable and Clean Energy) and SDG 12 (Responsible Consumption and Production), as it contributes to resource inefficiency and environmental strain. In contrast, the biological method of delinting aligns with several SDGs. Utilizing microorganisms or enzymes in the process tends to be more environmentally friendly, reducing the need for harsh chemicals and excessive energy. This supports SDG 13 (Climate Action) by mitigating the carbon footprint associated with traditional delinting methods. Additionally, the biological approach is often more socially sustainable, promoting healthier working conditions and aligning with SDG 8 (Decent Work and Economic Growth). By choosing biological delinting, we contribute to a more sustainable and responsible approach to cotton processing, actively supporting multiple SDGs.

## Conclusion

In conclusion, this review underscores the significance of cotton, elucidates its composition, and comprehensively evaluates various lint removal methods. While mechanical and chemical methods exhibit their distinct advantages and drawbacks, a noteworthy emphasis is placed on the biological method of delinting. This eco-friendly approach not only aligns with sustainable agricultural practices but also contributes to broader socio-economic goals, promoting the wellbeing of farming communities. By fostering the adoption of biological delinting methods, we can advance our commitment to Sustainable Development Goals (SDGs), particularly those related to environmental sustainability, responsible consumption, and community prosperity. The integration of biological delinting holds promise in steering the cotton industry towards a more sustainable and socially responsible future.
